# Multi-attribute fuzzy pattern decision making based on information systems

**DOI:** 10.1038/s41598-023-43753-z

**Published:** 2023-09-30

**Authors:** Zhenduo Sun, Xiangzhi Kong

**Affiliations:** School of Science, Jiangnan University, Wuxi, 214122 China

**Keywords:** Applied mathematics, Information technology, Scientific data

## Abstract

This paper introduces an innovative approach aimed at enhancing multi-attribute decision-making through the utilization of fuzzy pattern recognition, with a specific emphasis on engaging decision-makers more effectively. The methodology establishes a multi-attribute fuzzy pattern recognition model within a hybrid information system framework. It categorizes attributes into natural and abstract groups, standardizes them, and employs membership functions to transform them into degrees of membership. This adaptable approach permits the derivation of various decision criteria from the hybrid system. Subsequently, a testing set is generated from this system, and a suitable fuzzy operator is selected. The optimal solution is determined by assessing the similarity between the standard and testing sets. To underscore its effectiveness, a practical example is provided. Crucially, in the realm of multi-attribute decision-making, our method simplifies the process by reducing computational steps in contrast to the conventional TOPSIS model, while maintaining consistent outcomes. This streamlines the decision-making process and reduces complexity. We also demonstrate its applicability in multi-objective decision-making through a case study evaluating exemplary educators, thereby highlighting its adaptability and effectiveness. This method exhibits significant promise for enhancing multi-attribute decision-making and offers practical applications.

## Introduction

The emergence of multi-attribute fuzzy pattern decision-making is an eloquent response to the intricate quandaries presented by uncertainty, notably encompassing the pervasive haze of ambiguity woven into the tapestry of decision-making processes. In the realm of daily existence, the versatile tool of fuzzy language often finds its application to encapsulate the nebulous facets inherent in various phenomena. In such intricate settings, decision-making experts recurrently harness fuzzy language as a means to scrutinize divergent solutions, a strategic recourse driven by their inherent cognitive confines and subjective predilections.

However, the journey to effective multi-attribute fuzzy pattern decision-making has encountered methodological intricacies. Dong^1^ proffered a methodology pivoting on the fuzzy number vertex method to grapple with such complexities; however, its pragmatic feasibility has been marred by its inherent intricacy. The landscape of weight determination within the realm of multi-attribute fuzzy pattern decision-making has witnessed the inception of various strategies, encompassing the grayscale analysis method, the augmented fuzzy weighted average, and the upgraded fuzzy weighted average. The harmonious fusion of fuzzy numbers with the foundational bedrock of original multi-attribute decision-making paradigms has engendered enhanced congruence between theoretical constructs and practical exigencies. Noteworthy iterations of fuzzy numbers encompass intuitionistic triangular fuzzy numbers, intuitionistic trapezoidal fuzzy numbers, and Pythagorean fuzzy numbers.

Yue^2^ introduced a group decision model entrenched in the bastion of intuitionistic fuzzy numbers, adeptly amalgamating objective values while simultaneously streamlining the decision-making process. Concomitantly, Yi et al.^3^ ingeniously unveiled a three-branch decision model steeped in the edifice of Pythagorean fuzzy theory, a feat that encompassed the optimization of scoring functions and the novel instantiation of an action utility function for risk assessment. Subsequent contributions emanated from Zhang et al.^4^, who conceived a multi-attribute decision-making methodology scaffolded on the tenets of intuitionistic trapezoidal fuzzy numbers. This intricate stratagem encompassed the calculus of distances between trapezoidal fuzzy numbers, attribute weight determination via entropy and grayscale analysis, and the harmonious amalgamation of positive and negative ideal solutions in the framework of TOPSIS for the computation of grayscale relational projection values, culminating in the selection of the optimal alternative.

Concurrently, scholars such as Atanassov et al.^5^ embarked upon refining decision-making procedures through the strategic augmentation and extension of generalized net models. In a similar vein, Kacprzyk et al.^5^ furnished a robust framework encapsulating networks and fuzzy systems, alongside an auxiliary technique for inter-criteria analysis involving a three-dimensional exponential matrix. The visionary enterprise of Deng et al.^6^ manifested in the proposal of adversarial game decision-making within the realm of a fuzzy decision environment, this being facilitated through the utilization of fuzzy contradictory theory to model the intricate fabric of decision-making interactions. Meanwhile, the underpinning methodologies underwent evolution, an evolution epitomized by Zheng et al.^7^ who adroitly interwove trust relationships into a multi-attribute decision-making framework. The perennial conundrum of curtailing preference reversal within the precincts of the TOPSIS scheme became the focal point of intervention by Wang et al.^8^.

A retrospective glance into the 1970s unfurls the inception of the hierarchical analysis method^9^, a pivotal approach that methodically juxtaposes attribute merits and demerits across each tier to judiciously select a solution. Meanwhile, the entropy–TOPSIS method^10,11^ stands as a preeminent paradigm for multi-attribute decision assessment, enjoying wide-ranging applications across diverse industrial and agricultural spheres. Nonetheless, the actual domain of decision-making is characterized by a plethora of reference points and the enigma of pervasive fuzziness, compelling the expeditious assimilation of fuzzy sets^12^ and rough sets^13^ within the precincts of decision science. In the arena of fuzzy decision making, the harmonious symbiosis of fuzzy numbers^14–22^ with the scaffolding of original multi-attribute decision-making methodologies has engendered heightened compatibility between theoretical constructs and practical exigencies.

Within this intricate realm of information systems, Yao's three-branch decision method^23–27^ has ascended to prominence as a method of choice for the extraction of decision rules. This method, steering clear of conventional binary decision paradigms, introduces the novel dimension of delayed decision-making dynamics, aligning more astutely with the intricate nuances that typify human decision behavior. Notwithstanding, decision methods predicated on binary relations often bestow a solitary decision spectrum, circumscribed by the selection of binary relations. Notably, these information system-rooted decision methodologies, while seminal, at times grapple with the intricate task of effectively ranking solutions within the labyrinthine contours of multi-attribute decision-making. Regarding the latest multi-attribute decision-making methods, there are several studies concerning the application of multi-attribute decision-ranking methods^28–30^. In recent years, the application of fuzzy sets and information systems in multi-attribute decision making—although increasingly saturated—still has a certain degree of relevance^31–37^. The application of fuzzy pattern recognition in the field of medicine^38^ or in combination with fuzzy numbers^39^ for the study of intuitionistic fuzzy sets and fuzzy pattern recognition^40^ is also very popular. In addition, fuzzy pattern recognition is also applied in water quality identification^41^.

With the rapid advancement of information and computer technology, pattern recognition plays a pivotal role in the field of artificial intelligence. Fuzzy pattern recognition, in particular, finds extensive application in the domain of assessment. However, in the context of multi-attribute fuzzy pattern decision-making, the specific application of fuzzy pattern recognition remains underexplored. Concurrently, driven by the progress and development of modern technology, the trajectory of the multi-attribute decision-making process is shifting from a purely rational decision-making paradigm towards a more reference-based approach, grounded in the preferences of decision-makers. The increasingly intertwined relationship between behavioral decision-making and superiority underscores this evolution.

To transcend this landscape, this study adopts an innovative approach by integrating fuzzy pattern recognition into information systems. Through this approach, decision-makers' preferences and requirements are integrated into the process of establishing criterion sets and attributing weights, enhancing the fidelity to decision-makers’ actual needs. Additionally, due to the flexibility inherent in selecting the standard set, adjustments can be made in line with decision-makers' varying requirements, thereby rendering the overall decision-making process more adaptive and pliable. In contrast to traditional multi-attribute fuzzy pattern decision-making methods, the approach employed in this study, based on fuzzy pattern recognition, demonstrates enhanced efficiency and conciseness. It eliminates the need to determine positive and negative ideal solutions based on available information, requiring only the identification of a standard set and the selection of the same membership function to compute the set of interest. As a result, the computational process becomes more streamlined and efficient. Furthermore, the calculated results based on the fuzzy pattern recognition model are more reliable and accurate, thus furnishing decision-makers with increased confidence in their decision-making foundation.

In summation, this study introduces fuzzy pattern recognition into the realm of multi-attribute decision-making, effecting innovative enhancements to the decision-making process by aligning it more closely with actual requirements and bolstering its efficiency and reliability. This innovation not only expands the application scope of multi-attribute decision-making methods but also furnishes decision-makers with optimized and intelligent decision support.

This paper consists of four parts: Section “Introduction” presents the background of the investigation, Section “Preliminaries” provides preparatory knowledge, Section “Information system-based multi-attribute fuzzy pattern decision model” provides the algorithmic model, and Section “Example of an algorithm” provides a comparative calculation example with the traditional multi-attribute decision-making method TOPSIS to verify the model’s feasibility and superiority. An example of a company selecting talent is applied to verify the feasibility of multi-objective decision making.

## Preliminaries

### Fuzzy set operation rules

#### Definition 1 (Ref.^2^)

*Let *$U$* be a universe. A fuzzy set *$\widetilde{A}$* or a fuzzy subset *$\widetilde{A}$* of *$U$* is defined by a function that assigns each element *$x$* of *$U$* to a value *$\widetilde{A}\left(x\right)\in \left[0, 1\right].$* We use *$F \left(U\right)$* to denote the family of all fuzzy subsets of *$U$*, i.e., the set of all functions from *$U$* to *$[0, 1]$*, which is called the fuzzy power set of* $U.$

Let there be two fuzzy sets $\widetilde{A}$ and $\widetilde{B}$ in a universe $U$ with affiliation functions of ${\mu }_{\widetilde{A}}$ and ${\mu }_{\widetilde{B}}$, respectively. Then, the merge, intersection, and complement operations for fuzzy sets are defined as follows:

Merge: $${\mu }_{\widetilde{A}\cup \widetilde{B}}\left(u\right)={\mu }_{\widetilde{A}}\left(u\right)\bigvee {\mu }_{\widetilde{B}}\left(u\right)=\mathit{max}\left({\mu }_{\widetilde{A}}\left(u\right),{\mu }_{\widetilde{B}}\left(u\right)\right)$$;

Intersection: $${\mu }_{\widetilde{A}\cap \widetilde{B}} \left(u\right)= {\mu }_{\widetilde{A}}\left(u\right)\bigwedge {\mu }_{\widetilde{B}} \left(u\right)=\mathit{min}\left({\mu }_{\widetilde{A}} \left(u\right), {\mu }_{\widetilde{B}}\left(u\right)\right)$$;

Complement: $${\mu }_{\widetilde{A}}^{c}\left(u\right)=1-{\mu }_{\widetilde{A}}\left(u\right)$$.

### Principles of fuzzy pattern recognition

#### Definition 2 (Ref.^44^)

*Let* $F\left(U\right)$ *be a fuzzy power set of the universe *$U$*; *$\widetilde{A},\widetilde{B},\widetilde{C}$* are all fuzzy subsets of *$F\left(U\right)$*. If the mapping* $\sigma :F\left(U\right)\times F\left(U\right)\to \left[0, 1\right]$ *satisfies*

Normalization: $$\sigma \left(\widetilde{A}, \widetilde{A}\right)=1, \sigma \left(U, *\right)=0$$;

Symmetry: $$\sigma \left(\widetilde{A},\widetilde{B}\right)=\sigma \left(\widetilde{B}, \widetilde{A}\right), \forall \widetilde{A},\widetilde{B}\in U$$;

Inequality: $\widetilde{A}\subseteq \widetilde{B}\subseteq \widetilde{C}\to \sigma \left(\widetilde{A},\widetilde{C}\right)\le \sigma \left(\widetilde{A},\widetilde{B}\right)<\sigma \left(\widetilde{B},\widetilde{C}\right)$, then $\sigma \left(\widetilde{A},\widetilde{B}\right)$ is called the closeness of $\widetilde{B}$ to $\widetilde{A}$. $\sigma$ is defined as the closeness function on $F\left(U\right)$.

#### Definition 3 (Ref.^45^)

*Let* $\widetilde{{A}_{1}}, \widetilde{{A}_{2}}, \cdots \widetilde{, {A}_{n}}$ *be n fuzzy sets on a universe* $U$ *, and* $\widetilde{B}$ *be an object to be identified on* $U$ *; if*

$$\sigma \left(\widetilde{B}, \widetilde{{A}_{i}}\right)=max\left(\sigma \left(\widetilde{B},{\widetilde{A}}_{1}\right),\sigma \left(\widetilde{B},{\widetilde{A}}_{2}\right),\cdots ,\sigma \left(\widetilde{B},{\widetilde{A}}_{n}\right)\right)$$

*then we say that* $\widetilde{B}\in \widetilde{{A}_{i}}$ *.* $\sigma$ *is defined as the closeness function on* $F\left(U\right)$ *.*

Note: This definition is the principle of maximum affiliation in fuzzy pattern recognition.

#### Definition 4 (Ref.^45^)

Consider a universe $U$ and any mapping ${\mu }_{\widetilde{A}}$ from $U$ to the closed interval $\left[0, 1\right]$*.*

$$\left\{\begin{array}{c}{\mu }_{\widetilde{A}}: U\to \left[0, 1\right]\\ u\to {\mu }_{\widetilde{A}}\left(u\right)\end{array}\right.$$

Both determine $\widetilde{A}$, the fuzzy subset of $A$ of $U. {\mu }_{\widetilde{A}}$ becomes the membership function of the fuzzy subset, ${\mu }_{\widetilde{A}}\left(u\right)$ is called the membership degree of u to $\widetilde{A}$, and a fuzzy subset is called a fuzzy set if there is no misunderstanding.

### Fuzzy pattern recognition steps

The steps of fuzzy pattern recognition are given below^41^:

*Step 1* Select the set of characteristic factors of the pattern $X={\{x}_{1}, {x}_{2}\cdots {x}_{n}\}$; each object ${x}_{j}$ has m sample attributes that make up the set of ${x}_{j}={\left({x}_{1j}, {x}_{2j},\cdots {,x}_{mj}\right)}^{T}$; this forms the matrix of the measured attributes $X=\left({x}_{ij} \right)\left(i =1, 2\cdots ,m;j=1, 2\cdots ,n\right)$;

*Step 2* Classify attributes m by level c criteria model, then we have the attribute criteria matrix. $Y={\left({y}_{ih}\right)}_{m\times c}\left(2\le c<m\right)$;

*Step 3* Use the normalization formula to eliminate the influence of the physical dimension of different attribute characteristics and normalize characteristic values. We obtain the relative affiliation of the attributes of the sample to be identified ${r}_{ij}\left(i =1, 2,\cdots ,m;j=1, 2,\cdots ,n\right)$ to obtain the relative affiliation matrix of the attributes of the sample to be identified $R={\left({r}_{ij}\right)}_{m\times n}, 0\le r\le 1\left(i=1, 2, \cdots ,m;j=1, 2, \cdots ,n\right)$;

*Step 4* Similar to Step 3, we obtain the relative affiliation of each standard sample attribute ${S}_{ih}\left(i =1, 2,\cdots ,m;h=1, 2,\cdots ,c\right)$ and, similarly, obtain the relative affiliation matrix $S={\left( {s}_{ih}\right)}_{m\times c}$, $0\le {s}_{ih}\le 1, \left(i =1, 2\cdots ,m;h=1, 2\cdots ,c\right)$;

*Step 5* Determine the attribute weights ${\omega }_{ij}$, which are generally determined by the entropy weighting method or expert weighting method;

*Step 6* Construct a theoretical model for fuzzy pattern recognition, and construct a fuzzy pattern recognition matrix $U={\left({u}_{ij}\right)}_{c\times n}$. ${u}_{ij}$=$f\left(\omega , {S}_{ih}, {r}_{ij}\right)$;

*Step 7* Calculate the comprehensive evaluation index ${\theta }_{i}$, ($i =1, 2,\cdots ,m)$, which is the closeness of the solution. Then rank the best solution according to the principle of monological proximity.

### Commonly used closeness formula

Hamming distance^38^

1 $${\sigma }_{1}\left(\widetilde{A},\widetilde{B}\right)=1-\frac{1}{n}{\sum }_{k=1}^{n}\left|\widetilde{A}\left({x}_{k}\right)-\widetilde{B}\left({x}_{k}\right)\right|.$$

Euclidean distance^38^

2 $${\sigma }_{2}(\widetilde{A},\widetilde{B})=1-\frac{1}{\sqrt{n}}\left(\left[\sum_{k=1}^{n}\left(\widetilde{A}\left({x}_{k}\right)-\widetilde{B}\left({x}_{k}\right)\right)^{2}\right]\right)^\frac{1}{2}.$$

### Information system

#### Definition 5 (Ref.^25^)

Reference () *Call* $IS=\left\{U, AT, V, f\right\}$ *an information system, where *$U$* is a non-empty finite set of objects, *$U=\left\{{x}_{1}, {x}_{2}, \cdots , {x}_{q}\right\}$*; denote the set of attributes as *$AT$*; *$f$* is an information function, and *$\forall a\in AT$*, *$x\in U,$* we have *$f\left(x, a\right)\in {V}_{a}$*.*

On this basis, a multi-attribute fuzzy pattern decision-making method based on information systems is proposed.

## Information system-based multi-attribute fuzzy pattern decision model

### Information system establishment

The attributes are classified according to the characteristics of the values of different attributes in the information system. All affiliation functions and values are included.

#### Definition 1

*A set of an information system *$GIS=\left\{U, AT, V, f,g,G\right\}$* is established based on the decision object, where *$U$* is a non-empty finite set of objects, *$U = \left\{{x}_{1},{x}_{2},\cdots ,{x}_{q}\right\}$*; the set of attributes is *$AT=N\cup AB$*, *${V=V}_{n}\cup {V}_{ab}$*;* ${V}_{n}$ and ${V}_{ab}$ are the value domains of natural and abstract attributes, respectively; $f$ is the information function. $\forall a\in AT$*, *$x\in U$*, we have *$f\left(x, a\right)\in {V}_{a}$*; *$g$* is the set of affiliation functions; *$G$* is the set of affiliation values, and *$\forall a\in AT$*, *$x\in U$*, we have *$g\left(f\left(x, a\right)\right)\in {G}_{a}$*.*

$N$ is a non-empty finite set of natural attributes, denoted as $N={\{n}_{1}, {n}_{2}\cdots , {n}_{k}\}$. $AB$ is defined as a non-empty set of abstract attributes, which are expressed as $AB={\{ab}_{1}, {ab}_{2}\cdots , {ab}_{l}\}$. Their attribute values are in a non-numeric form such as language. The number of elements in $g$ is the same as the number of elements in $AT$, as shown in Table 1.

**Table 1:** Improved information system GIS.

	${a}_{1}$	${a}_{2}$	$\cdots$	${a}_{l}$
${x}_{1}$	${f}_{1}\left({x}_{1}\right),{g}_{1}({f}_{1}\left({x}_{1}\right))$	${f}_{2}\left({x}_{1}\right),{g}_{2}({f}_{2}\left({x}_{1}\right))$	$\cdots$	${f}_{l}\left({x}_{1}\right),{g}_{l}({f}_{l}\left({x}_{1}\right))$
${x}_{2}$	${f}_{1}\left({x}_{2}\right),{g}_{1}({f}_{1}\left({x}_{2}\right))$	${f}_{2}\left({x}_{2}\right),{g}_{2}({f}_{2}\left({x}_{2}\right))$	$\cdots$	${f}_{l}\left({x}_{2}\right),{g}_{l}({f}_{l}\left({x}_{2}\right))$
$\vdots$	$\vdots$	$\vdots$	$\ddots$	$\vdots$
${x}_{n}$	${f}_{1}\left({x}_{n}\right),{g}_{1}(f\left({x}_{n}\right))$	${f}_{2}\left({x}_{n}\right),{g}_{2}({f}_{2}\left({x}_{n}\right))$	$\cdots$	${f}_{l}\left({x}_{n}\right),{g}_{l}({f}_{l}\left({x}_{n}\right))$

Compared with the traditional hybrid information system (Mixed Attribute Value Information System), the improved hybrid information system divides attributes by their value characteristics. This allows for separate data processing for different attribute value characteristics and improves data processing speed. In the process of selecting conditional and decision attributes, the improved hybrid information system can determine attributes according to the decision maker’s preferences, i.e., the selection of relatively rational natural attributes or abstract attributes that take into account the subjective opinions of decision makers. The decision maker’s preference is taken into account in the process of selecting decision-related and conditional attributes. The set of affiliation functions and the set of affiliation degrees are added to the hybrid information system. This facilitates the selection of the affiliation function after normalizing the attribute values and determining the associated values. Different affiliation functions exhibit different attribute characteristics, so the introduction of the affiliation function set helps the decision maker to express preference-related information.

The following is an example of how to structure the required information system based on existing information.

#### Example 1

*Two companies of different sizes in city A, *${A}_{1}$* and *${A}_{2}$*, have to choose a partner company for their city B strategy, and there are three companies in city B, *${B}_{1}$*, *${B}_{2}$*, and *${B}_{3}$*, for which they can choose. The relevant information is presented in *Tables 2 and 3* below.*

**Table 2:** Companies’ information in City B.

	${N}_{1}$	$A{B}_{1}$	${N}_{2}$	${N}_{3}$
${B}_{1}$	${n}_{11}$	$a{b}_{11}$	${n}_{12}$	${n}_{13}$
${B}_{2}$	${n}_{21}$	$a{b}_{21}$	${n}_{22}$	${n}_{23}$
${B}_{3}$	${n}_{31}$	$a{b}_{31}$	${n}_{32}$	${n}_{33}$

**Table 3:** Information sheet for real estate companies’ projects and renovation companies.

	${N}_{1}$	${N}_{2}$	$A{B}_{1}$	${N}_{3}$	${N}_{4}$
${A}_{1}$	${n}_{11}^{1}$	${n}_{21}^{1}$	$a{b}_{11}^{1}$	${n}_{31}^{1}$	${n}_{41}^{1}$
	${n}_{12}^{1}$	${n}_{22}^{1}$	$a{b}_{12}^{1}$	${n}_{32}^{1}$	${n}_{42}^{1}$
	${n}_{13}^{1}$	${n}_{23}^{1}$	$a{b}_{13}^{1}$	${n}_{33}^{1}$	${n}_{43}^{1}$
${A}_{2}$	${n}_{11}^{2}$	${n}_{21}^{2}$	$a{b}_{11}^{2}$	${n}_{31}^{2}$	${n}_{41}^{2}$
	${n}_{12}^{2}$	${n}_{22}^{2}$	$a{b}_{12}^{2}$	${n}_{32}^{2}$	${n}_{42}^{2}$
	${n}_{13}^{2}$	${n}_{23}^{2}$	$a{b}_{13}^{2}$	${n}_{33}^{2}$	${n}_{43}^{2}$

The information system $GIS$ is constructed according to Tables 2 and 3, where the domain $U=\left\{{B}_{1},{B}_{2},{B}_{3}\right\}$; the set of attributes $AT=\left\{{N}_{1},{N}_{2},A{B}_{1},{N}_{3}\right\}$, where the natural attribute $N=\left\{{N}_{1},{N}_{2},{N}_{3}\right\}$, and the abstract attribute $AB=\left\{A{B}_{1}\right\}$; the attribute values $V$ are the values corresponding to each attribute in Table 3. Set the set of affiliation functions to $g$.

### Establishment of the standard set and the set to be tested

The following algorithm is based on fuzzy pattern recognition theory as a decision method, which was first proposed in 1991 by Chinese scholar Chen, and has since been widely used in the field of agriculture^42^; however, the decision method has certain drawbacks and tends to adopt empiricism in the process of determining evaluation indicators, while the determination of attribute weights is also limited to expert-given or other subjective attribute determination methods. In addition, fuzzy pattern recognition can also perform multi-objective matching, i.e., set-to-set matching in addition to point-to-set matching, which is more suitable for multi-objective decision making. Both methods are applied in this algorithm for fuzzy pattern recognition.

*Step 1* Building the information system GIS.

If each attribute in $AB=\left\{a{b}_{1},a{b}_{2}\cdots ,a{b}_{l}\right\}$ is split into ${c}_{i}\left(i=\mathrm{1,2},\cdots n\right)$ levels, and the values of the abstract attribute are converted into constants between 0 and 1, then each level is divided as shown in Table 4.

**Table 4:** Division of abstract attribute levels.

	${ab}_{1}$	${ab}_{2}$	$\cdots$	${ab}_{l}$
${c}_{1}$	$\frac{1}{i}$	$\frac{1}{i}$	$\cdots$	$\frac{1}{i}$
${c}_{2}$	$\frac{2}{i}$	$\frac{2}{i}$	$\cdots$	$\frac{2}{i}$
$\vdots$	$\vdots$	$\vdots$	$\ddots$	$\vdots$
${c}_{i}$	1	1	$\cdots$	1

The value obtained after the division is used as the normalized value of the attribute.

*Step 2* Classify the objective function and find the weights.

The target attributes are selected from the attribute set and classified into attributes. The default target attribute is mixed, i.e., the natural attributes ${n}_{\tau },\tau =\left(\mathrm{1,2},\cdots ,k\right)$ and $a{b}_{\varepsilon },\varepsilon =\left(\mathrm{1,2},\cdots ,l\right).$ For abstract attributes, the normalized values are determined according to the attribute abstraction hierarchy in Step 1. To eliminate the influence of different physical scales, decision-making-normalized values are compared with the maximum values of the same attribute.

In the domain of a standard information system, there are $z$ attributes,$c$ attributes are selected as target attributes—each target attribute is used as a reference sequence, and the remaining $(z-c)$ attributes are used to calculate the correlation ${r}_{ij}\left(i=\mathrm{1,2},\cdots ,c,j=\mathrm{1,2},\cdots ,z-c\right)$ to the target attribute using correlation analysis—and the sum of the correlations ${R}_{i}$ and the influence weight ${\omega }_{ij}$ is obtained. Please refer to Table 5.

**Table 5:** Relative values of natural attributes after removing physical dimensions.

	${n}_{1}$	${n}_{2}$	$\cdots$	${n}_{k}$
${m}_{1}$	${\gamma }_{11}$	${\upgamma }_{12}$	$\cdots$	${\gamma }_{1k}$
${m}_{2}$	${\gamma }_{21}$	${\gamma }_{22}$	$\cdots$	${\gamma }_{2k}$
$\vdots$	$\vdots$	$\vdots$	$\ddots$	$\vdots$
${m}_{p}$	${\gamma }_{p1}$	${\gamma }_{p2}$	$\cdots$	${\gamma }_{pk}$

3 $${R}_{i}={\sum }_{j=1}^{z-c}{r}_{ij},$$

4 $${\upomega }_{ij}=\frac{{r}_{ij}}{{R}_{i}}.$$

The objective attributes are assigned weights using subjective or expert assignment methods so that the sum of the objective function’s attribute weights is 1.

*Step 3* Determine the criterion set by choosing the affiliation function.

To construct a fuzzy set with each target attribute and non-target attribute, all fuzzy sets are merged to obtain the criterion set $\widetilde{\left(BZ\right)}$. This can be used to satisfy the decision makers’ preferences. The criteria matrix $A$ is constructed by determining the criteria scheme from the target attribute weights.

There are $z$ attributes in the subject of a standard information system’s domain, and $c$ attributes are selected as target attributes. The relative affiliation of the target attribute is denoted by ${g}_{i}\left(x\right)=m{b}_{i}\left(i=\mathrm{1,2}\cdots ,c\right)$, and the relative affiliation of the non-target attribute is denoted by.

$${g}_{j}\left(x\right)=fm{b}_{j}\left(j=\mathrm{1,2},\cdots ,z-c\right).$$

The target attribute carries a weight ${\rho }_{i}\left(i=\mathrm{1,2}\cdots c\right)$, and satisfies,

5 $${\sum }_{i=1}^{c}{\rho }_{i}=1$$

and

6 $${\widetilde{p}}_{i}={\sum }_{j=1}^{z-c}{\sum }_{i=1}^{c}\left(\frac{m{b}_{i}}{{MB}_{i}}+\frac{{\omega }_{ij}fm{b}_{j}}{{FMB}_{j}}\right).$$

Thus, the standard set is ${\widetilde{p}}_{i}\in \widetilde{BZ}$, and the standard matrix is $A$. Example 2 is given below to verify the feasibility of Step 2.

#### Example 2

*Using the direct economic losses of disasters as the target attribute, the influence weight of other attributes was calculated, as shown in *Table 6*.*

**Table 6:** 2000–2003 disaster loss table.

Year	2000	2001	2002	2003
Direct economic losses (RMB billion)	2045.30	1942.20	1637.20	1884.20
Crop damage area (thousand hectares)	34,374.0	31,793.0	27,319.0	32,516.0
Earthquake disaster losses (RMB billion)	14.6792	14. 8449	1.47740	46.6040

The maximum value of the same attribute was selected and the physical dimension was eliminated to normalize the attribute value, shown in Table 7.

**Table 7:** Disaster loss table after eliminating physical dimension.

Year	2000	2001	2002	2003
Direct economic loss (RMB billion)	1.00	0.95	0.80	0.92
Crop damage area (thousand hectares)	1.00	0.92	0.79	0.95
Earthquake disaster losses (RMB billion)	0.31	0.32	0.03	1.00

From this, the correlation between the area of crop damage (thousand hectares) (${x}_{1}$) and earthquake damage (billion yuan) (${x}_{2}$) and the direct economic damage (billion yuan) (${x}_{3}$) was calculated using gray correlation analysis with correlation degrees of 0. 59 and 0. 48 to calculate the impact weight. Table 8 was obtained.

**Table 8:** The weights of different attributes affecting the direct economic loss resulting from disasters.

Attributes	Impact weights
Area of crop damage (thousand hectares)	0.55
Earthquake disaster damage (billion yuan)	0.45

Set each attribute affiliation function to ${g}_{i}\left(x\right)=\frac{{f}_{i}}{{f}_{max}}$.

The resulting set of criteria is constructed as

$$\widetilde{p}=\frac{0.5}{{x}_{1}}+\frac{0.19}{{x}_{2}}+\frac{0.92}{{x}_{3}},$$

and the standard matrix is

$$A=\left(\begin{array}{ccc}0.5& 0.19& 0.92\end{array}\right).$$

*Step 4* Constructing the set to be tested.

The standard subject and the subject to be tested have the same or similar domains, and the target attributes in the set to be tested are selected according to the target attributes in the standard set and classified.

For the abstract attributes among the target attributes, the values are normalized according to the attribute abstraction hierarchy in Step 2. For the natural attributes of the subject to be measured, the natural attribute with the largest value in the subject to be measured is selected as the basis, and the remaining attributes are compared to the value ${\delta }_{mq}\in \left[\mathrm{0,1}\right], m=\left(\mathrm{1,2},\cdots ,p\right), q=\left(\mathrm{1,2},\cdots ,k\right)$. It can be worthwhile to denote

7 $${H}_{m}=\frac{{\sum }_{m=1}^{p}{\delta }_{mq}}{p},$$

as a normalized value.

The influence weight ${\omega }_{ij}$ of the non-target attributes on the target attributes in Step 3 is used as the influence weight of the non-target attributes on the target attributes in the subject of the information system domain to be tested. The normalized values are fuzzified by choosing the same affiliation function as in the standard set. The weights of the target attributes in the criterion set are assigned, and the sum of the attribute weights is 1. A fuzzy set is constructed for each target attribute and non-target attribute, and all fuzzy sets are merged to obtain the criterion set $\widetilde{\left(DC\right)}$. This is the basis for satisfying the decision maker’s preferences. The target attribute weights are used to determine the solution to be tested, and the matrix $B$ is constructed.

There are $z$ attributes in the subject of an information system domain to be tested, and $c$ attributes are selected as target attributes, The relative affiliation of the target attribute is denoted by ${g}_{i}\left(x\right)=m{b}_{i}\left(i=\mathrm{1,2}\cdots ,c\right)$ and the relative affiliation of the non-target attribute is denoted by ${g}_{j}\left(x\right)=fm{b}_{j}\left(j=\mathrm{1,2}\cdots ,z-c\right)$, and the weight of the target attribute is ${\alpha }_{i}\left(i=\mathrm{1,2}\cdots ,c\right)$, which satisfies

8 $${\sum }_{i=1}^{c}{\alpha }_{i}=1,$$

and

9 $$\widetilde{{y}_{i}}= {\sum }_{j=1}^{z-c}{\sum }_{i=1}^{c}\left(\frac{m{b}_{i}}{{MB}_{i}}+\frac{{\omega }_{ij}fm{b}_{j}}{{FMB}_{j}}\right).$$

Thus, the set to be measured is ${\widetilde{y}}_{\mathrm{i}}{\alpha }_{i}\in \widetilde{DC}$, and the matrix to be measured is $B$.

*Step 5* Selecting the fuzzy pattern recognition criterion and choosing the optimal solution.

The combined attribute value of two fuzzy vectors calculated by the closeness formula $\sigma (A,B)$ is called the closeness of these two fuzzy vectors.

*Step 6* Information system-based multi-attribute fuzzy pattern decision-making process.

**Figure 1:**
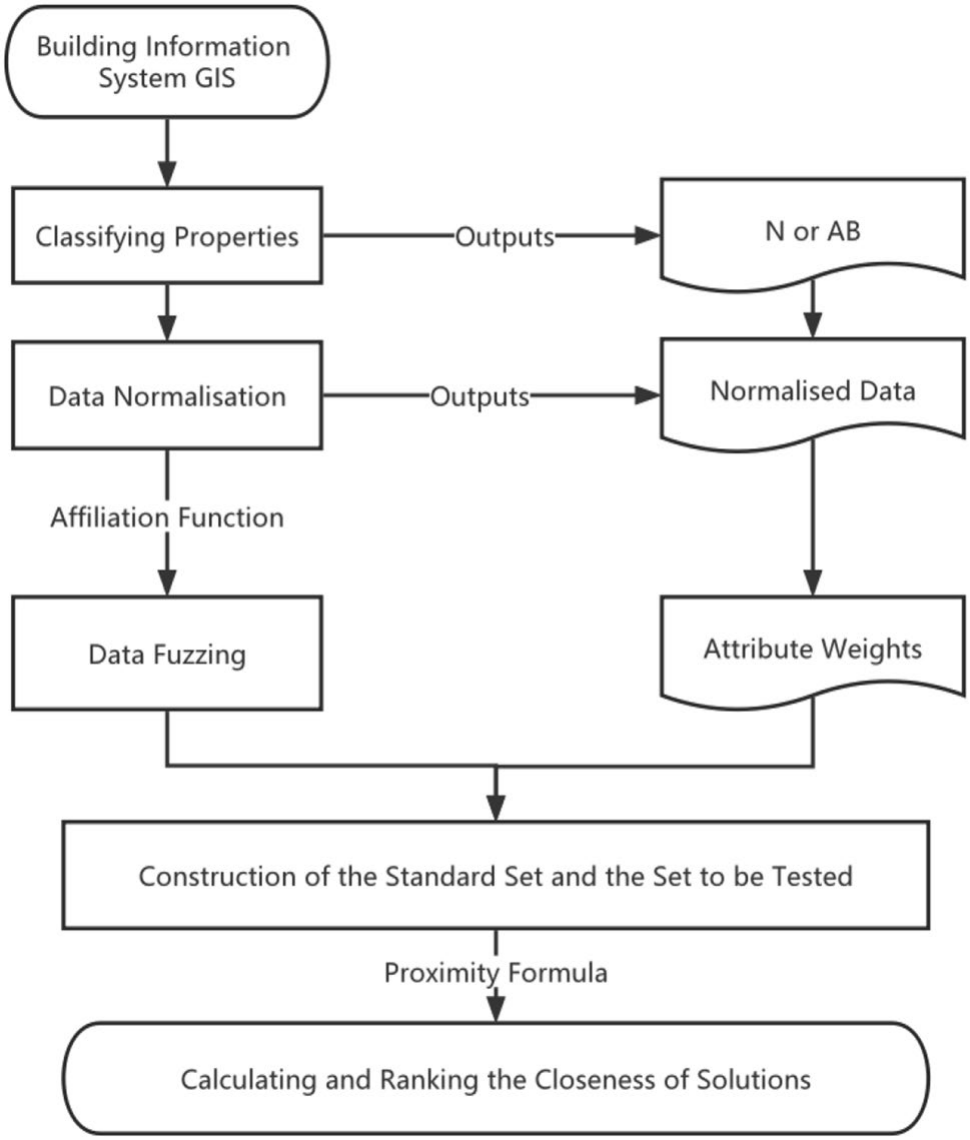
Multi-attribute decision-making process based on information system and fuzzy pattern recognition.

A table of steps and a flowchart of the information system-based multi-attribute fuzzy pattern decision-making process is given below (Table 9 and Fig. 1).

**Table 9:**
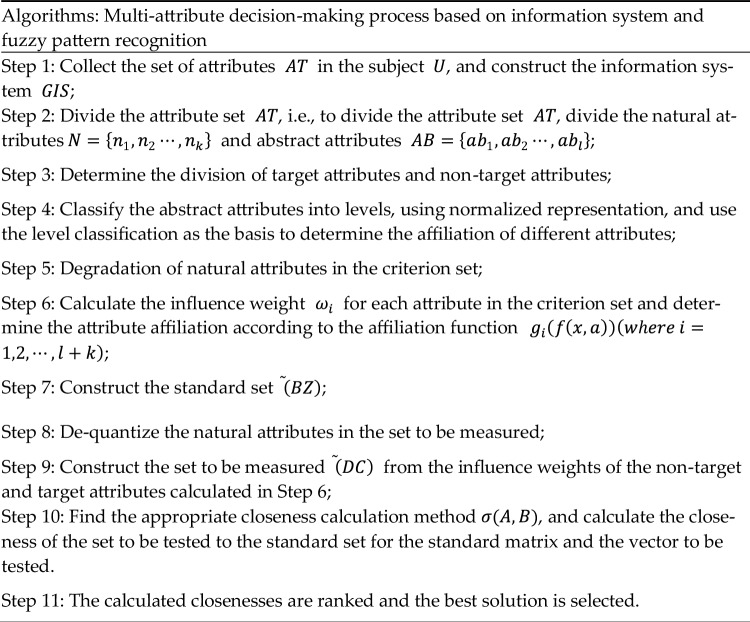
Multi-attribute decision-making process based on information system and fuzzy pattern recognition.

## Example of an algorithm

The correlation method used in this example is gray correlation analysis. The first step is to introduce the gray correlation analysis, which is the degree of influence of different factors on a particular factor in a gray system. The essence of the idea is to determine whether a series of curves is closely related to each other based on their similarity in geometry, as shown in Table 10.

**Table 10:**
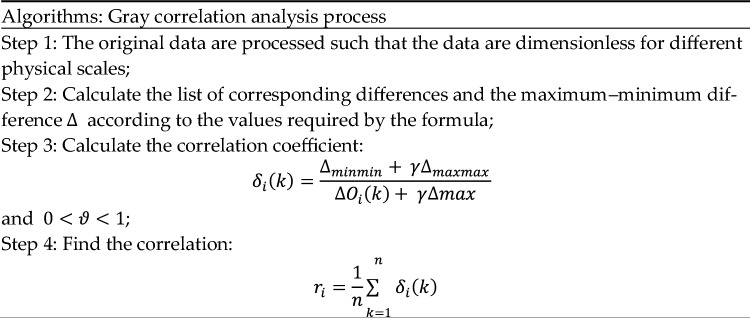
Gray correlation analysis process.

To demonstrate the advantage of the multi-attribute decision method, a comparative example with the TOPSIS method is given below. The TOPSIS algorithm cannot handle mixed-attribute problems, so a conventional TOPSIS example is chosen.

### Example 3

*Comparative example between the proposed method and the TOPSIS method.*

Among Table 11, ${a}_{1}$ and ${a}_{2}$ have an inverse effect on the selection result and ${a}_{3}$ has a positive effect on the selection result, that is, ${a}_{1}$ and ${a}_{2}$ are negatively related to the decision result and ${a}_{3}$ is positively related to the decision result.

**Table 11:** Decision information.

	${a}_{1}$	${a}_{2}$	${a}_{3}$
$A$	50.8	4.3	8.7
$B$	200.0	4.9	7.2
$C$	71.4	2.5	5.0
$D$	10.2	2.4	0.3

The TOPSIS method was used to select the best solution among five subjects. The data are normalized to obtain the following Table 12.

**Table 12:** Standardized attribute values.

	${a}_{1}$	${a}_{2}$	${a}_{3}$
$A$	0.1937	0.3281	0.0342
$B$	0.0492	0.2879	0.0413
$C$	0.1378	0.5643	0.0594
$D$	0.9648	0.5879	0.9907

In assigning weights to each attribute, the same weights are given in this case; thus, the matrix after the assignment is the same as the data in Table 12 used to select the optimal solution.

Then, we select the positive and negative ideal solutions based on the available data:

$${Z}^{+}=\left(0.9648\, 0.5879 \,0.9907\right)$$

$${Z}^{-}=\left(0.0492 \,0.2879\, 0.0342\right)$$

Calculating the distance between different solutions and positive and negative ideal solutions by the Formula (10), we obtain the following Table 13.

**Table 13:** Distances between different schemes and positive and negative ideal solutions.

	${{\varvec{D}}}^{+}$	${{\varvec{D}}}^{-}$
$A$	1.2558	0.1465
$B$	1.3527	0.0071
$C$	1.2457	0.2913
$D$	0.0000	1.3577

10 $$\left\{\begin{array}{c}{D}_{i}^{+}=\sqrt{{\sum }_{j=1}^{n}{\left({Z}_{ij}-{Z}_{j}^{+}\right)}^{2}}\\ {D}_{i}^{-}=\sqrt{{\sum }_{j=1}^{n}{\left({Z}_{ij}-{Z}_{j}^{-}\right)}^{2}}\end{array}\right.$$

In Table 14, the combined evaluation values of the different options are given and ranked with the Formula (11). A higher reference value corresponds to a better evaluation result.

**Table 14:** Combined evaluation value and ranking of different solutions.

Programs	Combined evaluation value	Rankings
$A$	0.1045	3
$B$	0.0052	4
$C$	0.1895	2
$D$	1.0000	1

11 $$\sigma ={D}_{worst}/\left({D}_{best}+{D}_{wors}\right)$$

The following calculation is performed using the method mentioned in this paper.

Construct a GIS information system where $U=\{A,B,C,D\}$ and $AT=\{{a}_{1},{a}_{2},{a}_{3}\}$; given $g={\{g}_{1}\}$, $V={f}_{i}\left(x,a\right)\left(i =\mathrm{1,2},3\right)$, where ${g}_{1}$ is defined as in Formula (12).

12 $$g_{1} = \left\{ {\begin{array}{*{20}c} 1 & {\left( {x > b} \right)} \\ {\left( {x - a} \right)/b - a} & {\left( {a \le x \le b} \right)} \\ 0 & {\left( {x < a} \right)} \\ \end{array} } \right..$$

After careful consideration, it was determined that the resulting information table does not have decision attributes, i.e., it is a decision problem aimed at choosing the most solutions. Therefore, all attributes are conditional attributes.

To ensure the comparability effect, the standardized attribute values are selected to be calculated directly as the affiliation degree, while the same weight is assigned to all attributes. The positive ideal solution found with TOPSIS is selected as the standard set, and the Hemming proximity is used to calculate the proximity between different solutions for the standard set, shown in Table 15 below.

**Table 15:** Program proximity and sequencing.

Programs	Program proximity	Rankings
$A$	0.3375	3
$B$	0.2733	4
$C$	0.4060	2
$D$	1.0000	1

Comparing the two methods, the same conclusions were reached and the validity of the scheme was verified.

We can find that the method provided in this paper is more concise in terms of the calculation process when the standardization conditions and weights are the same. Further, the selection of the control criteria provided in this paper is more flexible. The criteria set can be determined directly based on the needs of decision makers, which improves the participation of decision makers.

The following uses the choice of the bias affiliation function to compare the effects on the decision results. The calculated affiliation matrix is obtained as ${A}_{1}$.

$${A}_{1}=\left(\begin{array}{ccc}0.1578& 0.1340& 0.0000\\ 0.0000& 0.0000& 0.0074\\ 0.0968& 0.9213& 0.0263\\ 1.0000& 1.0000& 1.0000\end{array}\right)$$

After calculating the program posting schedule, we obtain Table 16.

**Table 16:** ${g}_{1}$ Program proximity and sequencing.

Programs	Program proximity	Rankings
$A$	0.0973	3
$B$	0.0025	4
$C$	0.3481	2
$D$	1.0000	1

By comparing Table 15 with Table 16, it is clear that by choosing different affiliation functions to classify the data characteristics, ${g}_{1}$ belongs to the biased large affiliation function, making the differences between the different schemes more obvious.

If the decision maker places extra importance on an attribute, the attribute can be considered a “secondary decision attribute” and the weight is calculated by associating the remaining attributes with it, as demonstrated in the following example.

Select ${\mathrm{a}}_{1}$ as the “secondary decision attribute” and calculate the association degree ${R}_{i}$ between ${a}_{2}$, ${a}_{3}$, and ${a}_{1}$, obtaining Table 17.

**Table 17:** Degree of correlation between attributes.

	${a}_{2}$	${a}_{3}$
${a}_{1}$	0.4620	0.8205

Calculating the weight division of different attributes yields Table 18.

**Table 18:** The weight of each attribute with ${a}_{1}$ as the “secondary decision attribute”.

	${a}_{1}$	${a}_{2}$	${a}_{3}$
$\omega$	0.4381	0.3595	0.2024

In this way, the affiliation function matrix ${A}_{2}$ is obtained by combining the affiliation matrix ${A}_{1}$ with the weights.

$${A}_{2}=\left(\begin{array}{ccc}0.0693& 0.0482& 0.0000\\ 0.0000& 0.0000& 0.0015\\ 0.0424& 0.3312& 0.0053\\ 0.4381& 0.3595& 0.2024\end{array}\right)$$

Option $D$ is chosen as the standard set, and the calculation is performed using the Hemming proximity to obtain the ranking results as shown in Table 19.

**Table 19:** Proximity and sorting after selecting “Sub-Decision Properties”.

Programs	Program proximity	Rankings
$A$	0.7057	3
$B$	0.6677	4
$C$	0.7930	2
$D$	1.0000	1

By comparing Tables 16 and 19, we find that after the decision maker selects the preferred attribute, it can be set as a “sub-decision attribute” and the weights can be calculated by grayscale correlation analysis to emphasize the decision maker’s preference while considering the interaction between different attributes. Although the decision result does not change, the change in the proximity can be seen; after choosing ${a}_{1}$ as “this decision attribute”, the difference between options $A$, $B$, and $C$ is reduced, and the decision maker’s preference is well-expressed.

An example is given to verify a simple application of the method in multi-objective decision-making. The example in question pertains a decision-making problem of a hybrid information system.

### Example 4

Two departments of a school, A and B, are recruiting in the job market. There are five applicants, and department A plans to recruit one member, and department B also plans to recruit one member. Relevant information is listed in Tables 20 and 21.

**Table 20:** Recruitment of departments A and B in the past 3 years.

	Recruiters	Interview results	Written test results	Work experience	Communication skills
Department A	$A$	90	85	2 years	Excellent
	$B$	92	83	3 years	Excellent
	$C$	95	83	5 years	Excellent
Department B	${A}_{1}$	80	100	1 year	Qualified
	${B}_{1}$	83	96	4 years	Medium
	${C}_{1}$	90	92	2 years	Good

**Table 21:** Information of five candidates to be hired.

	Interview results	Written test results	Work experience	Communication skills
Number 1	90	90	1 year	Good
Number 2	84	99	1 year	Medium
Number 3	85	96	2 years	Medium
Number 4	98	83	4 years	Excellent
Number 5	78	98	3 years	Qualified

To construct an information system based on Tables 20 and 21, we consider the following: domain $U$= {accepted person}, $AT$= {interview score $\left({x}_{1}\right)$,written test score $\left({x}_{2}\right)$, work experience $\left({x}_{3}\right)$, communication skills $\left({x}_{4}\right)$}. Now, we distinguish between the set of natural attributes $N$ = {interview scores $\left({x}_{1}\right),$ written scores $\left({x}_{2}\right)$} and the set of abstract attributes $AB$ = {work experience $\left({x}_{3}\right)$, communication skills $\left({x}_{4}\right)$} based on the characteristics of the attributes in the argument domain $U$.

The target attributes for department $A$ are interview performance $\left({x}_{1}\right)$ and communication skills $\left({x}_{4}\right)$; for department $B$, the target attributes are written test performance $\left({x}_{2}\right)$ and work experience $\left({x}_{3}\right)$. Given an affiliation function ${g}_{i}=\frac{{f}_{i}}{{f}_{max}}$ for each attribute, the affiliation values are the same as those obtained after normalization in Table 14; the abstract attributes are ranked as shown in Table 22 below. The results of de-quantizing all attributes are given in Table 23.

**Table 22:** Abstract Attribute Hierarchy.

Work experience	Grades	Communication skills	Grades
1 year	0.20	Excellent	1.00
2 years	0.40	Good	0.75
3 years	0.60	Medium	0.50
4 years	0.80	Qualified	0.25
5 years	1.00

**Table 23:** Hiring information obtained after de-quantizing.

	Recruiters	Interview results	Written test results	Work experience	Communication skills
Department A	$A$	0.95	1.00	0.40	1.00
	$B$	0.97	0.98	0.60	1.00
	$C$	1.00	0.98	1.00	1.00
Department B	${A}_{1}$	0.89	1.00	0.20	0.25
	${B}_{1}$	0.92	0.96	0.80	0.50
	${C}_{1}$	1.00	0.92	0.40	0.75

The results of using gray correlation analysis to obtain the weight of non-target attributes on target attributes in sector $A$ are shown in Table 24.

**Table 24:** Remaining attributes in department $A$ affect the weight of target attributes.

	Written test results	Work experience
Interview results	0.61	0.39
Communication skills	0.62	0.38

The interview results and communication skills are given equal weight in the target attributes, both 0.5. We combined these weights with the fuzzy concentration algorithm to construct a set of criteria for hiring personnel in department $A$ as follows:

$${\widetilde{p}}_{1}=\frac{0.97}{{x}_{1}}+\frac{0.61}{{x}_{2}}+\frac{0.26}{{x}_{3}}+\frac{1.00}{{x}_{4}}$$

The non-target attributes in department B are weighted against the target attributes as shown in Table 25.

**Table 25:** Influence weight of remaining attributes in department B on target attributes.

	Interview results	Communication skills
Written test results	0.59	0.41
Work experience	0.43	0.57

Written test score and communication ability are given the same weight in the target attribute, both being 0.5. We combined these with the fuzzy set algorithm to construct the criteria set of hiring personnel in department $B$ as

$${\widetilde{p}}_{2}=\frac{0.55}{{x}_{1}}+\frac{0.96}{{x}_{2}}+\frac{0.47}{{x}_{3}}+\frac{0.27}{{x}_{4}}$$

From this, write the standard matrix

$$P=\left(\begin{array}{cccc}0. 97& 0. 61& 0. 26& 1.00\\ 0. 55& 0. 96& 0. 47& 0. 27\end{array}\right)$$

In the following, we construct the set to be tested and de-quantized the information regarding the prospective employees, as shown in Table 26.

**Table 26:** Normalization of the information of the people to be hired.

	Interview results	Written test results	Work experience	Communication skills
Number 1	0.92	0.91	0.20	0.75
Number 2	0.86	1.00	0.20	0.50
Number 3	0.87	0.97	0.40	0.50
Number 4	1.00	0.84	0.80	1.00
Number 5	0.80	0.99	0.60	0.25

This was used to construct a matrix for each candidate.

$${A}_{1}=\left(\begin{array}{cccc}0.92& 0.55& 0.07& 0.75\\ 0.49& 0.91& 0.20& 0.28\end{array}\right)$$

$${A}_{2}=\left(\begin{array}{cccc}0.86& 0.52& 0.07& 0.50\\ 0.50& 1.00& 0.20& 0.21\end{array}\right)$$

$${A}_{3}=\left(\begin{array}{cccc}0.87& 0.51& 0.14& 0.50\\ 0.50& 0.97& 0.40& 0.20\end{array}\right)$$

$${A}_{4}=\left(\begin{array}{cccc}1.00& 0.50& 0.31& 1.00\\ 0.54& 0.84& 0.80& 0.46\end{array}\right)$$

$${A}_{5}=\left(\begin{array}{cccc}0.80& 0.48& 0.19& 0.25\\ 0.47& 0.99& 0.60& 0.10\end{array}\right)$$

**Figure 2:**
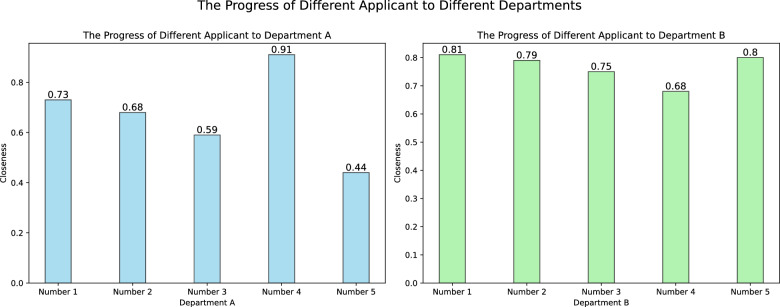
Histogram of approximate degree of applicability of candidates.

The Euclidean approximation is chosen for the standard set and the set to be tested, and the ranking depicted in Fig. 2 is obtained.

From Fig. 2 and Table 27, we can see that for department A: No. 4 > No. 1 > No. 2 > No. 3 > No. 5; for department B: No. 1 > No. 5 > No. 2 > No. 3 > No. 4.

**Table 27:** Ranking of candidates for departments A and B.

	Number 1	Number 2	Number 3	Number 4	Number 5
Department A	0. 73	0. 68	0. 59	0. 91	0. 44
Department B	0. 81	0. 79	0. 75	0. 68	0. 80

Therefore, it is recommended that department A hires candidate number 4 and department B hires candidate number 1.

From the above two examples, we can observe the following advantages of the model presented in this paper:

1. Compared to the traditional fuzzy pattern recognition methods mentioned in references 38–41, the approach presented in this paper integrates information systems with pattern recognition, reducing the number of attributes that may be overlooked in the decision-making process. It can also be adapted to rapidly evolving decision-making requirements. Furthermore, it combines information systems with attribute requirements and attribute value characteristics, enhancing the model's decision-making capability when dealing with mixed semantics in information systems.
2. The method employed in this paper calculates attribute weights using replaceable correlation analysis. In contrast to the weight calculation methods based on information entropy mentioned in references 13 and 23, this paper allows for the modification of weight measurement and determination methods based on different decision-making conditions, offering greater flexibility in determining conditional attribute weights. Additionally, it takes into account the subjective opinions of decision-makers. Decision attributes and conditional attributes are identified within the original attributes. The model constructs a fuzzy pattern recognition scheme for decision attributes. The calculated proximity value represents a comprehensive evaluation, considering both decision attributes and conditional attributes. Compared to TOPSIS, the calculation process in this paper is significantly simplified.
3. In comparison to the traditional three-branch decision-making processes discussed in references 24–27, this paper utilizes information systems as decision information carriers. However, it does not employ rough set theory to construct decision rules. Instead, it adopts an approach similar to machine learning, transforming information from the information system into fuzzy sets that can be used to calculate proximity. Additionally, this method draws inspiration from AHP and TOPSIS decision-making techniques to rank the calculated solutions, thereby arriving at a decision.
4. The decision-making method presented in this paper offers greater functionality compared to the multi-attribute decision-making methods mentioned in references 4, 9, and 12. When there is only one decision attribute, the method proposed in this chapter can be used as a multi-attribute decision-making approach for handling mixed information. Furthermore, when the problem is transformed into a multi-objective decision, the methods outlined in this chapter also demonstrate problem-solving capabilities.

## Conclusion

This paper presents a pioneering approach that unites the domains of fuzzy pattern recognition and information systems, culminating in the innovative proposal of a fuzzy pattern decision method. This novel method represents an endeavor to chart a new course in multi-attribute decision-making. It intricately amalgamates the strengths of fuzzy pattern recognition and information systems, addressing the challenges associated with attribute selection in fuzzy pattern recognition while concurrently enabling the representation of decision-making-related quaternions as fuzzy subsets within information systems.

In contrast to conventional multi-attribute fuzzy pattern decision-making methods, this paradigm eliminates the need for constructing positive and negative fuzzy ideal solutions rooted in information system data. Instead, it establishes a standard set tailored to align with the decision maker's preferences. Notably, the method integrates decision makers into three pivotal aspects: criteria selection, attribute weight determination, and the affiliation function, accentuating the unique characteristics of attributes. The flexibility in selecting the affiliation function aligns with decision makers' subjective inclinations. Furthermore, the method accommodates decision maker preferences and needs without invoking behavioral decisions.

It is essential to acknowledge the limitations of this model. Notably, the selection of the affiliation function may, at times, rely on experiential judgments, suggesting room for improvement in this aspect. Additionally, the method's applicability is confined to specific decision environments.

Moreover, through a comparative analysis of examples, it becomes apparent that the model proposed in this paper has certain limitations and benefits from prior decision-making experience to enhance decision accuracy. In the absence of prior decision-making experience, the results may be more influenced by the decision-maker's subjective preferences, potentially leading to decisions that overlook objective facts. Therefore, the scope of application for the multi-attribute decision-making model presented in this paper should be decision scenarios that fully consider the subjective needs of the decision maker, assuming a certain level of decision-making experience.

For future refinement, the model could be extended to encompass incomplete information systems, resulting in a nuanced approach that combines fuzzy pattern recognition with incomplete information systems. The flexibility in criteria set selection could be leveraged to determine attribute weights through a fusion of tolerance relations and gray correlation analysis. Furthermore, attributes' significance and information completeness could collaborate to determine attribute weights in the test set, optimizing the utilization of available information and aligning with decision maker preferences within the context of incomplete information systems. This approach could be harmonized with the interplay between behavioral decisions and dominance, further reinforcing decision preferences. This innovative avenue suggests a trajectory for solving multi-attribute decision-making challenges within the realm of incomplete information systems.

Furthermore, the algorithm outlined in this paper could be enriched by introducing intuitionistic fuzzy numbers, enabling the construction of intuitionistic fuzzy sets. The proximity between intuitionistic fuzzy sets could serve as the foundation for decision-making, culminating in a comprehensive comparison with existing algorithms to assess the merits and drawbacks of each approach.

## Data Availability

All data generated or analyzed during this study are included within the published article.
